# Intramuscular stiffness distribution in anterior and posterior upper trapezius muscles in healthy young males

**DOI:** 10.3389/fspor.2024.1507207

**Published:** 2024-12-06

**Authors:** Kohei Sasaki, Naokazu Miyamoto

**Affiliations:** ^1^ Graduate School of Health and Sports Science, Juntendo University, Chiba, Japan; ^2^ Institute of Health and Sports Science & Medicine, Juntendo University, Chiba, Japan

**Keywords:** myofascial pain syndromes, trigger point, shear wave speed, elastography, ultrasound

## Abstract

**Introduction:**

Increased muscle stiffness in the upper trapezius has been suggested to be associated with cervical myofascial pain and myofascial trigger points (MTrP). Recently, efforts have been made to objectively detect MTrP using ultrasound shear wave elastography (SWE). However, there is no consensus on the relationship between muscle stiffness assessed by SWE and MTrP. This may be due to the possibility that muscle stiffness is not uniform even in the asymptomatic trapezius. The present study aimed to characterize passive muscle stiffness at the proximal, central, and distal sites of the anterior and posterior parts of the upper trapezius.

**Methods:**

Seventeen healthy young males without neck pain participated in the study. The upper trapezius was divided into anterior and posterior parts based on anatomical landmarks: the line between C6 and the lateral end of the clavicle was defined as the anterior part, while the line between C7 and the acromion angle was defined as the posterior part. Shear wave speed (SWS; an index of stiffness) was measured using ultrasound SWE at six sites in the anterior and posterior parts of the upper trapezius, at 25% (proximal), 50% (central), and 75% (distal) of the muscle belly length.

**Results:**

SWS in the anterior part was significantly higher at the proximal (*p* < 0.001) and distal (*p* < 0.001) sites than at the central site. In the posterior part, there was no significant difference in SWS between the proximal, central, and distal sites. Comparisons between the anterior and posterior parts showed no significant differences in SWS at the proximal (*p* = 0.147), central (*p* = 0.339), and distal sites (*p* = 0.051).

**Conclusions:**

The characteristics of passive stiffness distribution in the anterior and posterior parts of the upper trapezius have important implications with respect to the optimal location of the control point during MTrP detection. In particular, it may be preferable to set the control point for detecting MTrP in the transverse direction rather than in the fascicle direction, that is, to compare passive muscle stiffness at the same levels between the anterior and posterior parts.

## 1 Introduction

Myofascial pain syndrome (MPS) is a type of musculoskeletal pain syndrome caused by myofascial trigger points (MTrP) in skeletal muscles and occurs most frequently in the upper trapezius (1). MTrP are hypersensitive spots within taut bands of a skeletal muscle that cause pain on compression and referred pain. MTrP can be classified into active and latent MTrP (2); the former causes spontaneous pain, while the latter does not cause spontaneous pain but elicits pain upon palpation. Latent MTrP are clinically asymptomatic; however, repetitive muscle overuse, acute muscle overload, or repetitive minor muscle trauma can transform them into active MTrP that cause musculoskeletal pain (3). In addition, the presence of latent MTrP in the upper trapezius can result in altered shoulder girdle dynamics, leading to musculoskeletal disorders such as impingement syndrome and rotator cuff lesions (4, 5). Therefore, early detection of the MTrP in the upper trapezius is important for the management of musculoskeletal pain and disorders. Clinically, MTrP are diagnosed by palpation of the taut band; however, palpation is considered to lack consistent reproducibility (6). Moreover, due to the lack of established objective and reliable diagnostic criteria, they are often overlooked.

Recently, efforts have been made to detect MTrP objectively. Ultrasound shear wave elastography (SWE), which can noninvasively quantify local tissue stiffness (7), has received particular attention for the diagnosis of MTrP in the upper trapezius. The previous studies (8) passive muscle stiffness at the central point of the upper trapezius was measured using ultrasound SWE in healthy individuals and those with taut bands and MTrP. The study found that individuals with MTrP had significantly higher passive muscle stiffness compared to healthy individuals. Furthermore, one of the previous studies (9) found a positive correlation between passive muscle stiffness and pain scores in patients with cervical MPS. The previous findings suggest that increased passive muscle stiffness is associated with MPS. In contrast, another study (10) compared passive muscle stiffness at the latent or active MTrP detected by palpation with that at a control point (3 cm lateral to the MTrP) in the upper trapezius. They failed to find significant differences in passive muscle stiffness at the latent or active MTrP compared to the control point. However, based on the previous findings, it is difficult to conclude that ultrasound SWE cannot detect MTrP from asymptomatic sites within a muscle. In this context, a recent review indicated that there is still no consensus regarding the association between passive muscle stiffness and pain symptoms (including MTrP) in the upper trapezius (11). Additionally, passive muscle stiffness in the upper trapezius has been reported to be non-uniform along the fascicle direction (12). Collectively, it may be preferable to set a control point for detecting MTrP in the transverse direction rather than in the fascicle direction in the upper trapezius.

The upper trapezius is transversely divided into two compartments (13, 14): anterior (between the sixth cervical spinous process and the lateral end of the clavicle) and posterior (between the seventh cervical spinous process and the acromion). The origins, insertions, and actions of the fascicles differ between the anterior and posterior parts of the upper trapezius. However, most of the available information on the upper trapezius passive stiffness is limited to the posterior part (8, 12), and detailed information on the anterior part is lacking. Understanding the stiffness distribution within the anterior and posterior parts of the upper trapezius in healthy individuals can be useful in determining the optimal control point location. Therefore, the purpose of the present study was to characterize passive muscle stiffness at the proximal, central, and distal sites of the anterior and posterior parts of the upper trapezius. Based on previous findings of non-uniform stiffness distribution along the fascicle direction in the upper trapezius, we formulated the following hypotheses: the passive stiffness of both the anterior and posterior parts of the upper trapezius is not uniformly distributed along the fascial direction. Furthermore, we proposed that there is no difference in passive stiffness between the anterior and posterior (transverse) parts, allowing them to serve as control points when comparing with MTrP. Based on this assumption, we established an additional hypothesis: the passive stiffness of the proximal, central, and distal sites does not differ between the anterior and posterior parts.

## 2 Materials and methods

### 2.1 Subjects

Seventeen healthy young males (171 ± 5 cm, 66 ± 8 kg, 22 ± 3 years old) participated in the present study. The exclusion criteria were as follows: (1) taking pain medications, muscle relaxants, or steroids; (2) currently undergoing physical therapy; (3) having a history of diagnosis of orthopedic disorders (cervical osteoarthritis, cervical disc herniation, cervical spondylosis nerve root disease, cervical spondylosis myelopathy, scoliosis); (4) having a history of neck or shoulder injuries (whiplash); (5) having a history of surgery on the spine, chest, shoulder joint, or upper arm; (6) having a history of diagnosis of a medical disorder (diabetes, Ehlers-Danlos syndrome, autoimmune disease, cancer, infectious disease, or cerebrovascular disease); and (7) having skin lesions that interfere with ultrasound measurements of the upper trapezius. The Japanese version of the FLANDERS handedness Questionnaire (15) was used to assess the dominant hand. Subjects were asked to avoid strenuous exercises within 48 h prior to the experiment.

### 2.2 Experimental setup and procedure

All measurements were performed on the right side of each subject. The subjects lay prone on an examination bed with a face hole, with their arms resting alongside their trunk and forearms in a pronated position. The subject's face was placed on the face hole, and they were instructed to relax their muscles as much as possible. The lumbar and lower legs were firmly secured to the bed with nonelastic straps. In order to determine the measurement sites for ultrasound SWE and electromyography (EMG) prior to data acquisition, the spinous processes of the C6 and C7 vertebrae, the lateral end of the clavicle, and the acromion angle were first identified by palpation. In the present study, the lines between the C6 and the lateral end of the clavicle and between the C7 and the acromion angle were defined as the anterior and posterior parts of the trapezius, respectively. Ultrasound SWE and EMG measurements were performed at 25% (proximal), 50% (central), and 75% (distal) of the muscle belly length (determined by identifying the proximal and distal muscle-tendon junctions using B-mode ultrasonography) at each part. The measurement sites were marked on the skin with a waterproof pen. It is very difficult to perform ultrasound SWE and EMG measurements simultaneously because of insufficient surface area. Therefore, ultrasound SWE and EMG measurements were performed separately; ultrasound SWE measurement was followed by EMG measurement. Then, the presence of latent MTrP was investigated.

### 2.3 Ultrasound SWE measurement and analysis

**Figure 1:**
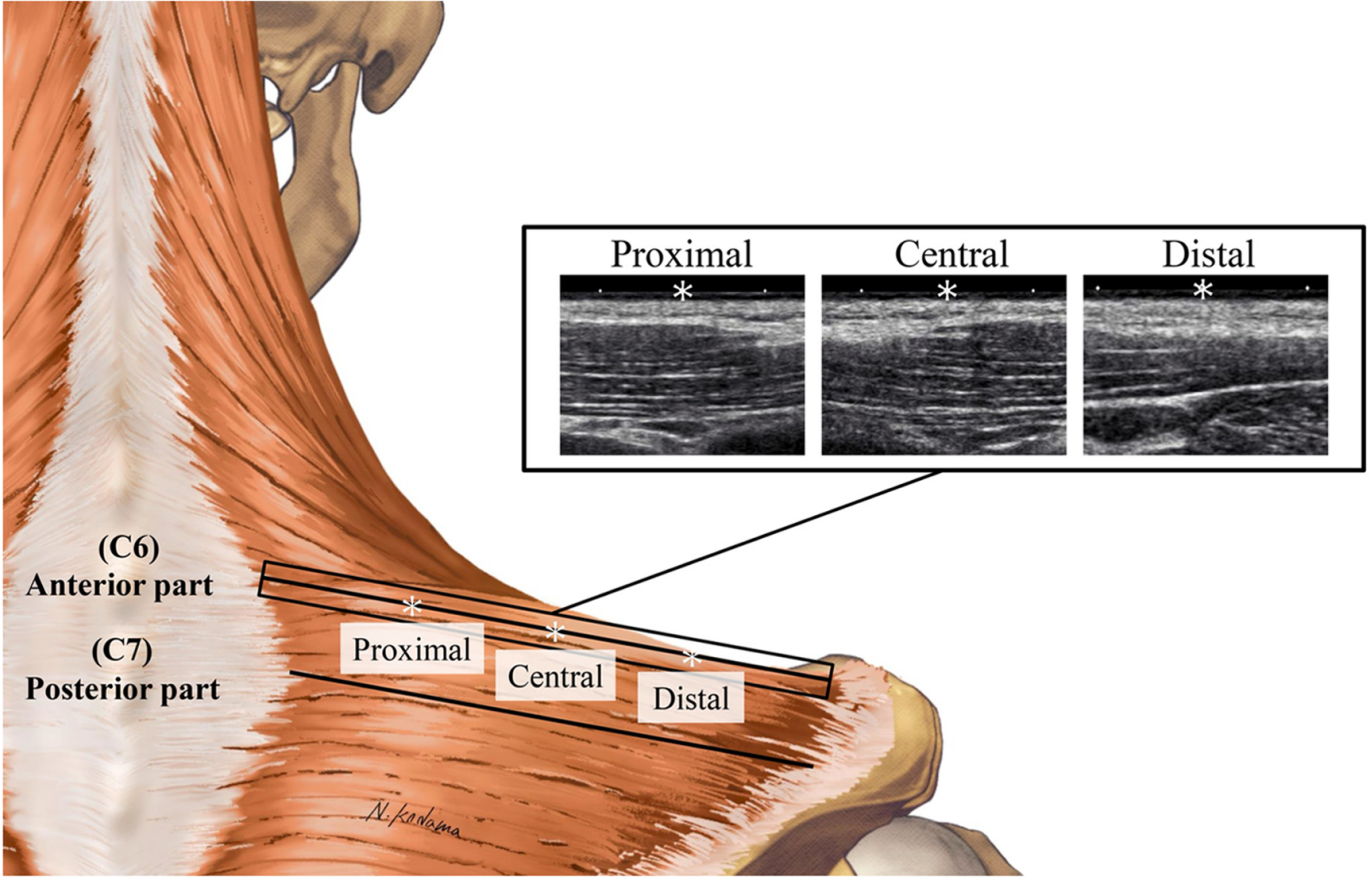
Examples of B-mode images at the proximal, central, and distal sites of the anterior part of the upper trapezius. * Denotes 25% (proximal), 50% (central), and 75% (distal) points of the muscle belly length.

**Figure 2:**
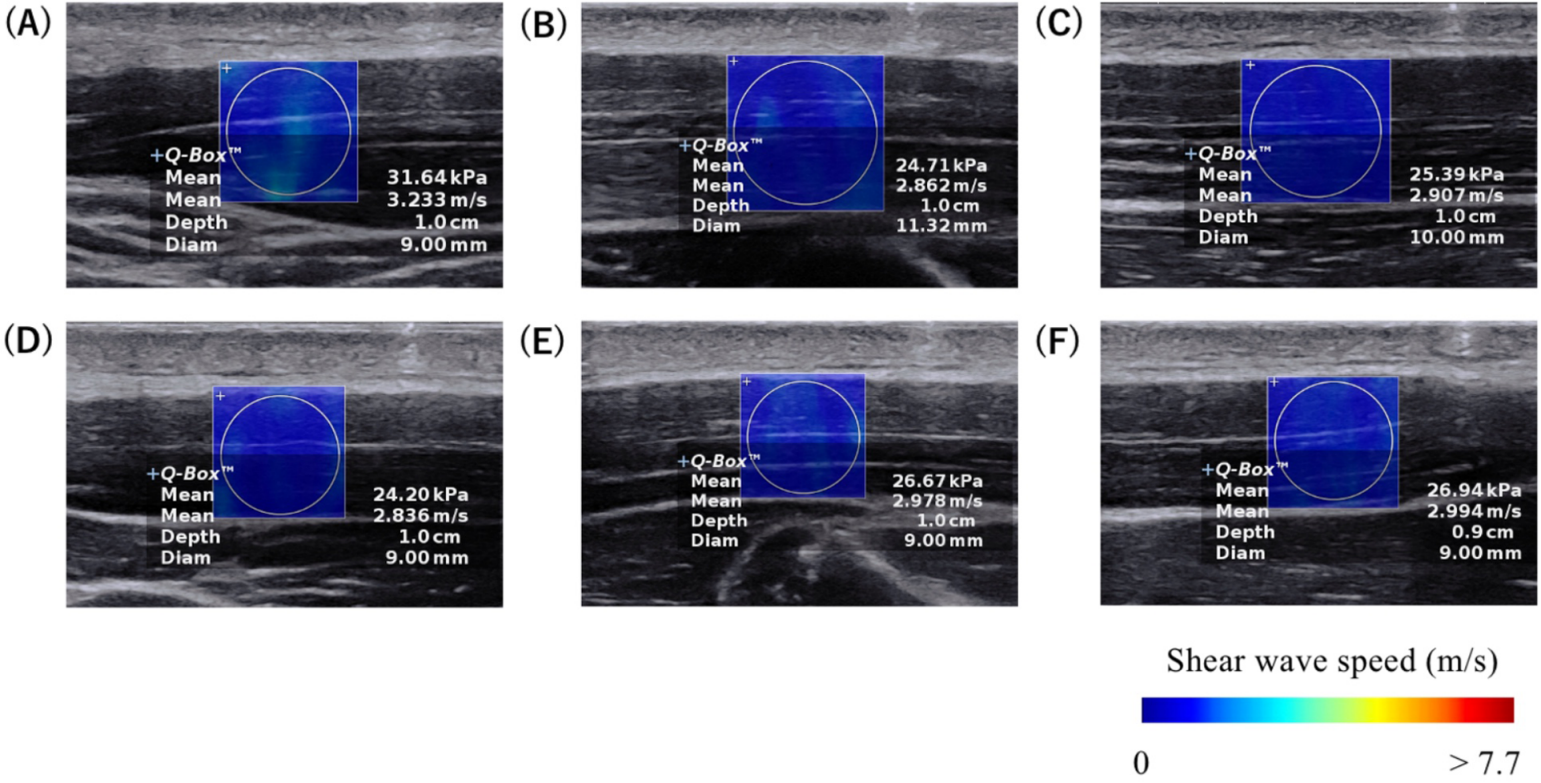
Typical examples of ultrasound SWE images at the **(A)** proximal, **(B)** central, and **(C)** distal sites of the anterior part, and at the **(D)** proximal, **(E)** central, and **(F)** distal sites of the posterior part of the upper trapezius. The colored region represents the shear wave speed map, with the scale shown at the bottom of the images.

An Ultrasound SWE system (Aixplorer Ver.12, Supersonic Imagine, France) with a 2–10 MHz linear probe (SL10-2, Supersonic Imagine, France) was used to assess muscle shear wave speed (SWS) (preset: “MSK”, persistence: high, smoothing: 5). The ultrasound probe was placed at the marked sites mentioned above, and the probe orientation was adjusted to identify several fascicles within the B-mode image (Figure 1). Care was taken not to press and deform the muscles while scanning. Ultrasound SWE images were acquired after confirming that the color map was stable for a few seconds. At each measurement site, three measurements were performed (i.e., three images were acquired). The Ultrasound SWE data were analyzed using software in the ultrasound SWE system. A circular area as large as possible, with the exclusion of fascia and subcutaneous fat tissue, was selected as the region of interest (Figure 2). The diameters of the regions of interest for each site are shown in Table 1. The mean SWS over the region of interest was calculated for each image. The values of three measurements at each site were averaged and used for statistical analyses.

**Table 1:** Diameter of region of interest in elastography measurement.

Upper trapezius	Proximal	Central	Distal
Anterior part (mm)	11.7 ± 1.6	9.6 ± 1.7	5.9 ± 1.5
Posterior part (mm)	7.9 ± 1.4	7.0 ± 1.8	5.7 ± 1.6

To evaluate the inter-trial (intra-day, intra-rater) reliability of ultrasound SWE measurements, a pilot study (*n* = 5) was performed prior to the main experiment mentioned above. The inter-trial reliability tests were performed three times with at least 5-min intervals by the same examiner who conducted the main experiment. In the reliability tests, the marks for the measurement sites were completely erased after each trial.

### 2.4 EMG measurement and analysis

Muscle activities of the upper trapezius were assessed at six measurement sites using a wireless surface EMG system (Trigno, Delsys, USA). Skin preparation that included shaving and cleaning with alcohol was performed before fixing the surface electrodes with adhesive tape at the marked sites. After the surface electrodes were fixed, EMG signals were recorded at 1 kHz using a 16-bit analog-to-digital converter (PowerLab/16SP, ADInstruments, Australia). Additionally, to normalize EMG data at rest, each subject performed one maximal voluntary isometric contraction (MVC) of shoulder abduction with the shoulder abducted at 90° against manual resistance in the prone position on the bed. During MVC, subjects were verbally encouraged. EMG data were processed using commercially available software (LabChart 8, ADInstruments, Australia). For each measurement site, the root-mean-square value of the EMG signal (EMG-RMS) for 5 s at rest was calculated and normalized to the maximal value of EMG-RMS during MVC.

### 2.5 Latent MTrP identification

The presence of the latent MTrP was tested in the prone position. A single examiner with more than 10 years of experience in the assessment and treatment of MTrP determined the presence or absence of latent MTrP, according to the diagnostic criteria by Fernández-de-las-Peñas et al. (16). The criteria for latent MTrP were as follows: (1) presence of a palpable taut band in the muscle; (2) presence of a hypersensitive tender spot in the taut band; and (3) local twitch response provoked by snapping palpation of the taut band. Among the measurement sites that met all three criteria, those where palpatory stimuli did not reproduce symptoms previously experienced by the subject, or where the evoked symptoms were not recognized by the subject as those previously felt, were considered latent MTrP (16). No latent MTrP was detected at any of the ultrasound SWE and EMG measurement sites.

### 2.6 Statistical analysis

The sample size was calculated using G*Power software (Kiel University, Germany). Since there were no previous data on the intramuscular difference in the upper trapezius stiffness to estimate the sample size, the minimum sample size was estimated with a “moderate” effect size for differences between measurement sites (*f*^2^ = 0.25), an α level of 0.05, and a power (1-β) of 0.8. According to our calculation, 14 subjects were required for an analysis of variance (ANOVA) with repeated measures. Thus, the 17 subjects in the present study satisfied the minimum sample size.

To evaluate the reliability of the ultrasound SWE measurements, intraclass correlation coefficient (ICC) (ICC1,3) and coefficient of variation (CV) were calculated using the pilot study data. The normality of the data distribution of SWS and EMG-RMS at each measurement site in the main experiment was assessed by the Shapiro-Wilk test. The SWS at the central site in the posterior part and EMG-RMS at the distal site in the anterior part and at the central and distal sites in the posterior part were not normally distributed. Therefore, the Friedman test was used to compare the variables between the proximal, central, and distal sites. When a significant main effect was detected, *post-hoc* Bonferroni-adjusted Wilcoxon signed-rank tests were performed. For comparisons between the anterior and posterior parts at the proximal, central, and distal sites, the Bonferroni-adjusted Wilcoxon signed-rank test was used. For all statistical tests, *p* < 0.05 was considered significant. The statistical analyses were performed with statistical software (SPSS statistics Ver. 29, IBM, USA).

## 3 Results

### 3.1 Subjects' characteristics

Among the 17 subjects, 16 were right-handed. Subjects had the following previous athletic experience (soccer [2], soft tennis [1], volleyball [1], powerlifting [1], cycling [1], baseball (outfielder) [3], and track and field [8]). The breakdown of the track and field events were sprint (2), mid-distance (1), long-distance (2), jump (2), and throw (1).

### 3.2 Reliability of measurement

For the SWS of the anterior part, ICC1,3 ranged from 0.79 to 0.91, with CV of 8.1% to 12.3%. For the SWS of the posterior part, ICC1,3 ranged from 0.82 to 0.83, with CV of 9.2% to 11.1%. ICC1,3 greater than 0.75 indicates good reliability.

### 3.3 SWS

**Figure 3:**
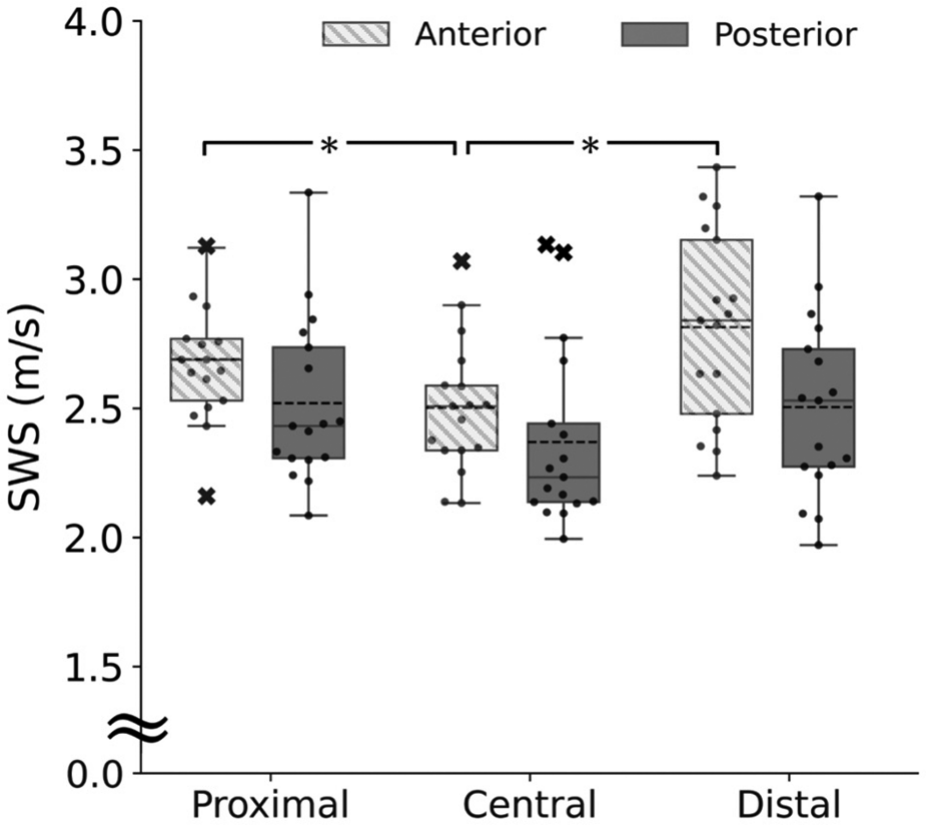
Upper trapezius shear wave speed (SWS) at the proximal, central, and distal sites of the anterior (box plot with shading) and posterior parts (gray). *Significant difference at *p* < 0.05.

Figure 3 shows the upper trapezius SWS at the proximal, central, and distal sites of the anterior and posterior parts. For the anterior part, the Friedman test showed a significant main effect (*p* < 0.001, observed power = 0.99). Follow-up *post-hoc* tests revealed that the SWS was significantly higher at the proximal (*p* < 0.001, observed power = 0.99) and distal sites (*p* < 0.001, observed power = 0.99) than at the central site. For the posterior part, the Friedman test did not show a significant main effect (*p* = 0.113, observed power = 0.76). For comparisons between the anterior and posterior parts, the Bonferroni-adjusted Wilcoxon signed-rank tests showed no significant difference at the proximal (*p* = 0.147, observed power = 0.95), central (*p* = 0.339, observed power = 0.81), and distal sites (*p* = 0.051, observed power = 0.99).

### 3.4 EMG-RMS

**Figure 4:**
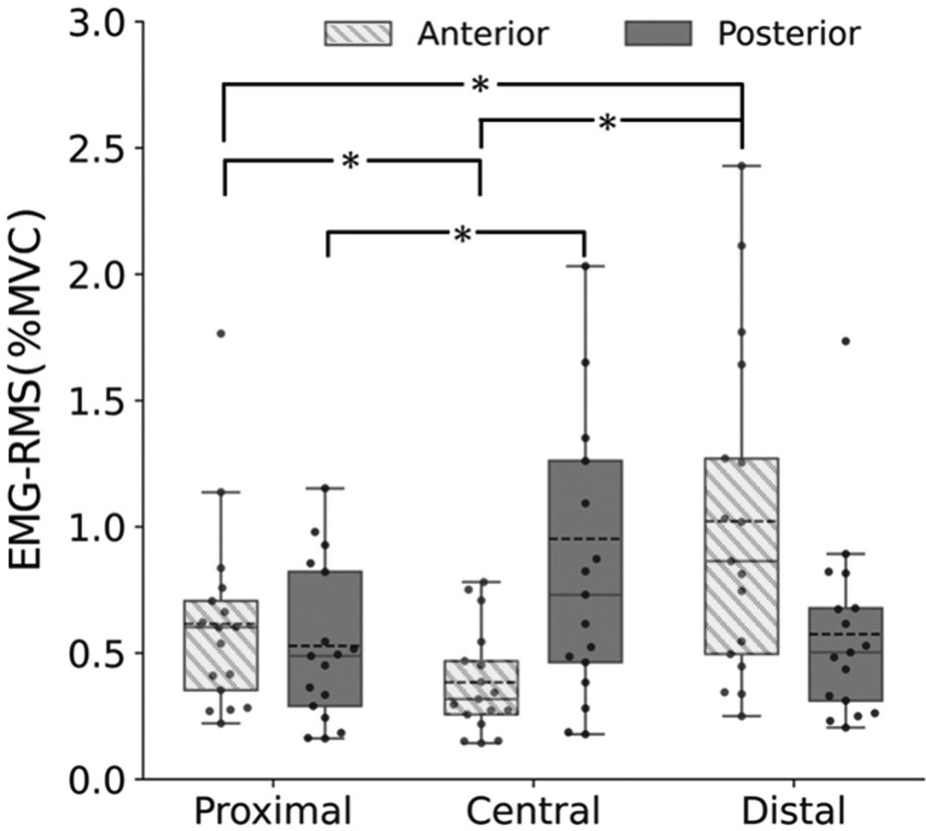
Upper trapezius muscle activities (EMG-RMS) at the proximal, central, and distal sites of the anterior (box plot with shading) and posterior parts (gray). *Significant difference at *p* < 0.05.

Figure 4 shows the EMG-RMS of the upper trapezius at the proximal, central, and distal sites of the anterior and posterior parts. For both the anterior and posterior parts, the Friedman test showed significant main effects (anterior: *p* < 0.001; posterior: *p* = 0.019). Follow-up *post-hoc* tests revealed that the EMG-RMS of the anterior part was significantly greater at the proximal (*p* = 0.049) and distal sites (*p* < 0.001) than at the central site and greater at the distal site than at the proximal site (*p* = 0.049). The EMG-RMS of the posterior part was significantly greater at the central site than at the proximal site (*p* = 0.018). The Bonferroni-adjusted Wilcoxon signed-rank test revealed that the EMG-RMS was significantly greater in the posterior part than in the anterior part at the central site (*p* < 0.001).

## 4 Discussion

The present study focused on the intramuscular distribution of passive stiffness in healthy subjects without latent MTrP. Multiple proximo-distal site SWS were measured using ultrasound SWE for the anterior and posterior parts of the upper trapezius. To our knowledge, this study is the first to demonstrate non-uniform intramuscular stiffness distribution in the anterior part of the upper trapezius.

In the present study, passive muscle stiffness in the anterior part of the upper trapezius was non-uniformly distributed, with stiffness at the proximal and distal sites being higher than at the central site. This result supports our hypothesis.

Previous studies have examined the intramuscular stiffness distribution in the hamstring (17) and gastrocnemius (18). In both muscles, passive muscle stiffness was in the order of the distal >central >proximal sites when measured in stretched positions, whereas passive muscle stiffness was uniform when measured in shortened (not stretched) positions. Our findings on the anterior part were not consistent with the previous findings, regardless of whether the measurement position in the present study was stretched or shortened. This discrepancy between the present and previous studies may be due, at least in part, to the difference in the muscles investigated, but it may also be due to the difference in the influence of muscle activity. The previous study investigating intramuscular stiffness distribution within the hamstring (17) showed that muscle activity at the distal site, where passive muscle stiffness was higher, was lower than at the proximal and central sites, concluding that the non-uniform distribution of passive muscle stiffness within the hamstring was not due to muscle activity. In contrast, in the present study, muscle activities were higher at the proximal and distal sites, where passive muscle stiffness was higher than at the central site. These findings suggest that the non-uniform distribution of passive muscle stiffness distribution in the anterior part of the upper trapezius may be due, at least in part, to the regional differences in muscle activity although absolute values of muscle activities were not large at either site.

Contrary to part of our hypothesis, passive muscle stiffness in the posterior part of the upper trapezius was uniformly distributed in the present study. In a previous study examining passive muscle stiffness distribution within the posterior part of the upper trapezius in healthy adults without shoulder pain and a history of neck or shoulder disorder, passive muscle stiffness was measured at four sites: 17%, 33%, 50%, and 67% of the line from the seventh cervical spinous process to the acromion (12). The highest passive muscle stiffness was observed at the 33% site, which is close to the proximal site of the present study (note that in the present study, the measurement sites were determined based on the muscle belly length rather than the origin-insertion length). The discrepant results between the present and previous studies may be due to differences in the measurement posture (prone in the present study and seated in the previous study) and the resulting degree of muscle stretching as mentioned above. On the other hand, although there were regional differences in muscle activities in the posterior part as observed in the anterior part, there were no significant differences in passive muscle stiffness. The reason for this is unknow at this time and requires further study.

This study is the first to compare the upper trapezius muscle stiffness between the anterior and posterior parts. Consistent with our hypothesis, we did not find significant differences in passive muscle stiffness between the two parts. Specifically, although a significant difference in muscle activity was found between the anterior and posterior parts at the central site, no significant difference in passive muscle stiffness was observed between these parts. The results of the present study on passive muscle stiffness have important implications for setting a control point for objective detection of MTrP using ultrasound SWE. Although there is no agreement on whether passive muscle stiffness at MTrP is higher than at normal sites, if MTrP exhibits higher passive muscle stiffness, setting the control point for MTrP detection in the fascicle direction may make MTrP detection difficult. For example, if the MTrP is located at the central site of the anterior part of the upper trapezius (where passive muscle stiffness is low in normal individuals, as in the present study) and the control point is set proximally or distally along the fascicle direction, the MTrP may be overlooked. Given the findings of the present study, it is preferable to set the control point for MTrP detection in the transverse direction rather than in the fascicle direction, that is, to compare passive muscle stiffness at the same levels between the anterior and posterior parts.

## 5 Limitations

The present study has some limitations. First, the present study included only healthy subjects without latent MTrP. Thus, it remains unclear whether the present findings hold for individuals with active or latent MTrP. Second, passive muscle stiffness of the upper trapezius in the present study was measured in the prone position. Therefore, it is unclear whether similar results would be obtained in other postures such as sitting. Third, all measurements in the present study were conducted on the right side. Although a consensus has not yet been reached regarding the influence of hand dominance on passive muscle stiffness of the upper trapezius, previous studies have reported both higher (19, 20) and lower (21) passive muscle stiffness in the upper trapezius on the dominant side. Given the possibility that hand dominance may have influenced the results of muscle stiffness distribution, further investigation on this point is needed. Fourth, a preliminary power analysis using pilot data was not conducted to determine the sample size in the present study. When a power analysis was conducted using the data obtained, with an alpha level of 0.05 and a power of 0.80, the minimum sample size required to detect differences within the anterior part and between the anterior and posterior parts at the proximal, central, and distal sites, was 17, indicating that the sample size of the present study met the criterion. In contrast, the minimum sample size required to detect differences within the posterior part was found to be 21. Therefore, some of the results should be interpreted with caution.

## 6 Conclusions

We investigated the intramuscular distribution of passive stiffness in the anterior and posterior parts of the upper trapezius in healthy adult males without MTrP. Muscle stiffness was non-uniformly distributed in the anterior part, whereas the posterior part showed a uniform distribution. There are no significant differences in muscle stiffness between the anterior and posterior parts. These results have important implications with respect to the optimal location of the control point during MTrP detection. In particular, it may be preferable to set the control point for detecting MTrP in the transverse direction rather than in the fascicle direction.

## Data Availability

The raw data supporting the conclusions of this article will be made available by the authors, without undue reservation.
